# The impact of dementia and language on hospitalizations: a retrospective cohort of long-term care residents

**DOI:** 10.1186/s12877-020-01806-2

**Published:** 2020-10-08

**Authors:** Karine Riad, Colleen Webber, Ricardo Batista, Michael Reaume, Emily Rhodes, Braden Knight, Denis Prud’homme, Peter Tanuseputro

**Affiliations:** 1 Faculty of Medicine, University of Ottawa, Ottawa, Canada; 2 Institut du Savoir Montfort, Ottawa, Canada; 3 Bruyère Research Institute, Ottawa, Canada; 4 Department of Medicine, Clinical Epidemiology Program, Ottawa Hospital Research Institute, 1053 Carling Ave, Ottawa, ON K1Y 4E9 Canada; 5 ICES, Ottawa, Canada; 6 Faculty of Health Sciences, University of Ottawa, Ottawa, Canada

**Keywords:** Dementia, Language, Long-term care, Hospitalizations, Nursing homes

## Abstract

**Background:**

Hospitalizations carry considerable risks for frail, elderly patients; this is especially true for patients with dementia, who are more likely to experience delirium, falls, functional decline, iatrogenic complications, and infections when compared to their peers without dementia. Since up to two thirds of patients in long-term care (LTC) facilities have dementia, there is interest in identifying factors associated with transitions from LTC facilities to hospitals. The purpose of this study was to investigate the association between dementia status and incidence of hospitalization among residents in LTC facilities in Ontario, Canada, and to determine whether this association is modified by linguistic factors.

**Methods:**

We used linked administrative databases to establish a prevalent cohort of 81,188 residents in 628 LTC facilities from April 1st 2014 to March 31, 2017. Diagnoses of dementia were identified with a previously validated algorithm; all other patient characteristics were obtained from in-person assessments. Residents’ primary language was coded as English or French; facility language (English or French) was determined using language designation status according to the *French Language Services Act*. We identified all hospitalizations within 3 months of the first assessment performed after April 1st 2014. We performed multivariate logistic regression analyses to determine the impact of dementia and resident language on the incidence of hospitalization; we also considered interactions between dementia and both resident language and resident-facility language discordance.

**Results:**

The odds of hospitalization were 39% lower for residents with dementia compared to residents without dementia (OR 0.61, 95% CI 0.57–0.65). Francophones had lower odds of hospitalization than Anglophones, but this difference was not statistically significant (OR 0.91, 95% CI 0.81–1.03). However, Francophones without dementia were significantly less likely to be hospitalized compared to Anglophones without dementia (OR 0.71, 95% CI 0.53–0.94). Resident-facility language discordance did not significantly affect hospitalizations.

**Conclusions:**

Residents in LTC facilities were generally less likely to be hospitalized if they had dementia, or if their primary language was French and they did not have dementia. These findings could be explained by differences in end-of-life care goals; however, they could also be the result of poor patient-provider communication.

## Background

Dementia is a disorder characterized by a decline in neurocognitive function that affects over 50 million people worldwide [1]. Individuals with dementia represent up two thirds of residents in long-term care (LTC) facilities [2], where they are more likely to experience poor functional and health outcomes when compared to their peers without dementia [3–5]. Studies have shown that healthcare transitions, such as transfers from LTC facilities to hospitals, carry considerable risks for LTC residents with dementia, such as delirium, falls, functional decline, iatrogenic complications, and infections [6–9]. As the number of individuals with dementia is expected to increase by 60% over the next 10 years [1], there is interest in identifying risk factors to prevent avoidable harm and unnecessary healthcare utilization among this population.

Communication is an integral component of the patient-provider relationship. Deficits associated with cognitive impairment (e.g., aphasia, confabulation) create communication barriers [10]; patients with dementia are more likely to have problems with comprehension of language, fluency of language, reading, and writing [11, 12]. Since health literacy requires a high level of cognition and communication, patients with dementia may be more likely to experience poor communication in healthcare settings.

Communication barriers can be exacerbated by language discordance [13], which occurs when a patient and their provider lack proficiency in a shared language. Language discordance can negatively impact access to healthcare services, quality of healthcare services, and patient outcomes [14, 15]. Previous studies have shown that residents who live in minority language situations face barriers when accessing healthcare services [16, 17], have longer emergency department visits and hospitals stays [18, 19], have higher rates of hospital admissions and re-admissions [20–22], and experience more harmful events in hospitals [23–27].

Previous studies have shown that LTC residents with dementia, compared to those without dementia, have lower rates of hospitalization [28–30]. Another study of LTC residents showed that Francophones in Ontario, Canada, were less likely to be hospitalized from language-concordant homes (i.e., French homes) than language-discordant homes (i.e., English homes) [31]. However, to our knowledge, the joint impact of dementia and language discordance on the risk of hospitalization for LTC residents has never been considered. The purpose of this study was to 1) compare the rate of hospitalization for residents with and without dementia in LTC facilities in Ontario, Canada, and 2) determine whether resident language and resident-facility language discordance modifies the relationship between dementia status and risk of hospitalization.

## Methods

### Study design and population

We conducted a population-based retrospective cohort study in Ontario, Canada, using linked administrative databases. We identified individuals who resided in LTC facilities from April 1, 2014 to March 31, 2017 through the Continuing Care Reporting System (CCRS), which is a database that captures information on individuals receiving continuing care services in hospitals, as well as individuals in LTC facilities that offer 24-h nursing services [2]. Data are collected using the Resident Assessment Instrument – Minimum Data Set 2.0 (RAI-MDS 2.0). Complete assessments are administered within 14 days of admission into LTC; they are also repeated annually and after significant changes in functional or health status. Abbreviated assessments are administered quarterly [2]. Residents who completed more than one assessment were indexed at the time of their first assessment in the study period and followed for 90 days. We excluded residents who: were younger than 65 or older than 105 at the time of the index assessment; or were not eligible for Ontario’s universal health insurance plan (i.e., the Ontario Health Insurance Plan (OHIP)) at any time during the study period.

### Data sources

Baseline characteristics, including functional and health characteristics as well as recent healthcare utilization, were obtained from the following databases: CCRS; OHIP claims database, which captures billing claims for healthcare services provided by physicians; Ontario Drug Benefit (ODB) database, which captures prescriptions dispensed to individuals who are eligible to receive publicly funded coverage of their prescription medications (i.e., residents over the age of 65 as well as residents living in LTC facilities); Registered Persons Database (RPDB), which captures personal information such as age, sex, and postal code. Finally, information related to hospitalizations were obtained from the Discharge Abstract Database (DAD), which captures information on all discharges from acute care hospitals. Chronic conditions were identified using algorithms validated by [removed for blinding] and applied in previous studies (Additional file 1) [32–41]. The datasets were linked using unique encoded identifiers and analyzed at [removed for blinding].

### Exposure

The following criteria were used to identify residents with dementia: 1) diagnosis of dementia during a previous hospitalization (obtained from the DAD), 2) three or more physician billing claims at least 30 days apart in a two-year period (obtained from the OHIP claims database), 3) prescription of a cholinesterase inhibitor (obtained from the ODB database), or 4) documentation of dementia or Alzheimer’s disease AND Cognitive Performance Scale score greater than or equal to 2 in index assessment or in any previous RAI assessment, including those administered for continuing care services (CCRS database) and home care services (RAI-HC database). The first three criteria of this algorithm have positive and negative predictive values of 80.4 and 99.0%, respectively, when applied to Ontario residents over the age of 65 [41]. The last criterion was added to increase the sensitivity of the algorithm.

Resident language was obtained from the index RAI assessment. During these assessments, interviewers are instructed to record the primary language spoken by the resident, which is coded as English, French, or other. Anglophones and Francophones were defined as residents whose primary spoken language was English and French, respectively, the two official languages of Canada. We excluded residents whose primary language was other than English or French. Language of the LTC facility was defined using language designation status according to the *French Language Services Act* [42], which is a provincial law that requires government agencies to provide all or some of their services in both English and French. In Ontario, 16 LTC facilities are included in this law; we defined these facilities as *French facilities* [43]. We defined the remaining 612 facilities, which are only required to provide services in English, as *English facilities*. Anglophones in French facilities and Francophones in English facilities were considered to be living in language-discordant facilities.

### Outcome

All hospital admissions within 90 days of each resident’s index assessment were identified from the DAD. The primary outcome was binary; we compared residents with at least one hospitalization (i.e., *hospitalized*) to residents without any hospitalizations (i.e., *not hospitalized*) during the 90-day follow-up period.

### Statistical analysis

We performed descriptive analyses to compare the characteristics of residents with and without dementia. We also compared outcomes after stratifying by dementia as well as language of the resident, language of the LTC facility, and resident-facility language discordance. Comparisons were performed using chi-square tests for categorical variables, analysis of variance (ANOVA) for normally distributed continuous variables, and the Mann-Whitney U test for ordinal variables.

The associations between dementia, resident language, facility language, and hospitalizations were further explored with multivariate logistic regression analyses. We ran three regression analyses; the first examined the relationship between dementia, resident language, and hospitalizations, while the second and third models considered resident language and resident-facility language discordance as effect modifiers of the association between dementia and hospitalization. In the first model, both dementia and resident language were included as independent covariates. The second model (dementia*language) included a categorical variable denoting the interaction between dementia status and resident language (i.e., Anglophone without dementia, Francophone without dementia, Anglophone with dementia, Francophone with dementia), and the third model (dementia*discordance) included a categorical variable denoting the interaction between dementia status and resident-facility language discordance (i.e., residents without dementia in language-concordant facilities, residents without dementia in language-discordant facilities, residents with dementia in language-concordant facilities, residents with dementia in language-discordant facilities). The reference categories consisted of Anglophones without dementia (model 2) and residents without dementia in language-concordant facilities (model 3). All regression models adjusted for potential confounders related to both resident characteristics (age, sex, education, urban/rural status of prior residence, number of prescription medications, Charlson Comorbidity Index [44], Changes in health, End stage disease, and Signs and Symptoms (CHESS) score [45], Activities of Daily Living scale [46]) as well as facility characteristics (income quintile of facility, urban/rural status of facility, total number of beds). Statistical tests were two-tailed, and the significance threshold was set at 0.05. We used SAS 9.3 (SAS Institute, Cary, NC) for all analyses.

## Results

We included a total of 81,188 LTC residents who met eligibility criteria. Residents with dementia represented 84.8% of the cohort. The baseline characteristics of the cohort are presented in Table 1. The proportion of female residents was greater among residents with dementia. Compared to residents without dementia, residents with dementia were generally older, more likely to have completed high school or university, and less likely to have lived in a rural area prior to entering LTC. Overall, 96.0% of residents were Anglophone and 97.7% of residents lived in English LTC facilities. Overall, the proportion of Francophones in English LTC facilities and Anglophones in French LTC facilities was 2.8 and 1.1%, respectively. Francophones (70.3%) were much more likely than Anglophones (1.1%) to live in language-discordant facilities. The proportion of Anglophones and Francophones did not differ across residents with and without dementia; however, the proportion of residents in English LTC facilities was greater for residents with dementia when compared to residents without dementia.

**Table 1:** Baseline characteristics of long-term care (LTC) residents in Ontario, stratified by dementia status

Baseline characteristics	Dementia (***N*** = 68,814)	No Dementia (***N*** = 12,374)	Total (***N*** = 81,188)	***P*** value
**Sex – no. (%)**				
Female	45,655 (66.3%)	7968 (64.4%)	53,623 (66.0%)	<.001
Male	23,159 (33.7%)	4406 (35.6%)	27,565 (34.0%)	
**Age (in years) +/− s.d.**	85.9 +/− 7.3	84.8 +/− 8.7	85.7 +/− 7.6	<.001
**Marital status – no. (%)**				
Not Married	28,528 (41.5%)	6496 (52.5%)	35,024 (43.1%)	<.001
Married	13,417 (19.5%)	2534 (20.5%)	15,951 (19.6%)	
Unknown	26,869 (39.0%)	3344 (27.0%)	30,213 (37.2%)	
**Education – no. (%)**				
Less than High School	18,387 (26.7%)	3440 (27.8%)	21,827 (26.9%)	<.001
High School	11,926 (17.3%)	1965 (15.9%)	13,891 (17.1%)	
Some Post-secondary	7269 (10.6%)	1319 (10.7%)	8588 (10.6%)	
University Graduate	4836 (7.0%)	780 (6.3%)	5616 (6.9%)	
Unknown	26,396 (38.4%)	4870 (39.4%)	31,266 (38.5%)	
**Urban/Rural Status of prior Residence – no. (%)**				
Urban	54,820 (79.7%)	9592 (77.5%)	64,412 (79.3%)	<.001
Rural	10,680 (15.5%)	2410 (19.5%)	13,090 (16.1%)	
Missing	3314 (4.8%)	372 (3.0%)	3686 (4.5%)	
**Language of Resident – no. (%)**				
English	66,014 (95.9%)	11,889 (96.1%)	77,903 (96.0%)	0.437
French	2800 (4.1%)	485 (3.9%)	3285 (4.0%)	
**Language of LTC Facility – no. (%)**				
English	67,304 (97.8%)	12,052 (97.4%)	79,356 (97.7%)	0.005
French	1510 (2.2%)	322 (2.6%)	1832 (2.3%)	
**Language concordance/discordance between resident and LTC Facility – no. (%)**				
Anglophone in English Facility	65,305 (94.9%)	11,741 (94.9%)	77,046 (94.9%)	0.004
Anglophone in French Facility	709 (1.0%)	148 (1.2%)	857 (1.1%)	
Francophone in English Facility	1999 (2.9%)	311 (2.5%)	2310 (2.8%)	
Francophone in French Facility	801 (1.2%)	174 (1.4%)	975 (1.2%)	

The functional status and health characteristics of the cohort are presented in Table 2. Residents with dementia tended to have fewer chronic conditions. (e.g., cancer, CHF, COPD, diabetes) and fewer hospitalizations in the 3 months preceding their entry into the cohort; they were also more likely to require total assistance with their activities of daily living. Moreover, residents with dementia experienced greater health decline (as determined by the CHESS score), and they were more likely to have a history of mental illness or developmental disability.

**Table 2:** Functional and health characteristics of long-term care (LTC) residents in Ontario, stratified by dementia status

Baseline characteristics	Dementia (***N*** = 68,814)	No Dementia (***N*** = 12,374)	Total (***N*** = 81,188)	***P*** value
**Number of Chronic Conditions – mean +/− s.d.**	3.90 +/− 2.03	4.80 +/− 2.04	4.04 +/− 2.06	<.001
**Chronic Conditions – no. (%)**				
Cancer	10,211 (14.8%)	3095 (25.0%)	13,306 (16.4%)	<.001
CHF	18,673 (27.1%)	5462 (44.1%)	24,135 (29.7%)	<.001
COPD	13,360 (19.4%)	3818 (30.9%)	17,178 (21.2%)	<.001
Diabetes	23,758 (34.5%)	5202 (42.0%)	28,960 (35.7%)	<.001
**History of mental illness or developmental disability – no. (%)**				
Yes	6972 (10.1%)	1099 (8.9%)	8071 (9.9%)	<.001
No	61,842 (89.9%)	11,275 (91.1%)	73,117 (90.1%)	
**Hospitalizations in last 90 days – no. (%)**				
0	54,889 (79.8%)	7414 (59.9%)	62,303 (76.7%)	<.001
1–2	13,548 (19.7%)	4729 (38.2%)	18,277 (22.5%)	
3–4	361 (0.5%)	220 (1.8%)	581 (0.7%)	
5+	16 (0.0%)	11 (0.1%)	27 (0.0%)	
**Prescription Medications – no. (%)**				
Less than 5	7113 (10.3%)	650 (5.3%)	7763 (9.6%)	<.001
5 to 9	29,116 (42.3%)	3531 (28.5%)	32,647 (40.2%)	
Greater than or equal to 10	32,585 (47.4%)	8193 (66.2%)	40,778 (50.2%)	
**Activities of Daily Living Scale – no. (%)**				
Independent	1671 (2.4%)	654 (5.3%)	2325 (2.9%)	<.001
Supervision Required	3070 (4.5%)	787 (6.4%)	3857 (4.8%)	
Limited Impairment	7712 (11.2%)	1706 (13.8%)	9418 (11.6%)	
Extensive Assistance Required (1)	20,167 (29.3%)	3085 (24.9%)	23,252 (28.6%)	
Extensive Assistance Required (2)	16,726 (24.3%)	3240 (26.2%)	19,966 (24.6%)	
Dependent	14,156 (20.6%)	2545 (20.6%)	16,701 (20.6%)	
Total Dependence	5302 (7.7%)	354 (2.9%)	5656 (7.0%)	
**CHESS score**				
No Health Instability	28,478 (41.4%)	4516 (36.5%)	32,994 (40.6%)	<.001
Minimal Health Instability	24,711 (35.9%)	4327 (35.0%)	29,038 (35.8%)	
Low Health Instability	11,161 (16.2%)	2470 (20.0%)	13,631 (16.8%)	
Moderate Health Instability	3199 (4.6%)	764 (6.2%)	3963 (4.9%)	
High Health Instability	1033 (1.5%)	235 (1.9%)	1268 (1.6%)	
Very High Health Instability	232 (0.3%)	62 (0.5%)	294 (0.4%)	

*ADL* Activities of Daily Living, *CHESS* Changes in Health, End-Stage Disease and Symptoms and Signs Scale, *CHF* Congestive Heart Failure, *COPD* Chronic Obstructive Pulmonary Disease

As shown in Table 3, residents with dementia were less likely to be hospitalized during the study period when compared to residents without dementia (10.5% vs. 20.3%; *p* < 0.001). The proportion of residents hospitalized was not associated with the language of the resident, the language of the LTC facility, or the resident-facility language discordance. However, we observed a non-significant decrease in hospitalizations for residents in language-concordant LTC facilities for both Anglophones (12.0 and 12.8% in English and French LTC facilities respectively) and Francophones (9.7 and 11.6% for Francophones in French and English LTC facilities, respectively).

**Table 3:** Hospitalizations of long-term care (LTC) residents within 3 months of assessment

	Hospitalized Within 3 Months (***N*** = 9718)	Not Hospitalized Within 3 Months (***N*** = 71,470)	***P*** value
**Dementia status – no. (% denotes row percentage)**			
No dementia	2509 (20.3%)	9865 (79.7%)	<.001
Dementia	7209 (10.5%)	61,605 (89.5%)	
**Language of Resident – no. (% denotes row percentage)**			
English	9354 (12.0%)	68,549 (88.0)	0.109
French	364 (11.1%)	2921 (88.9%)	
**Language of LTC Facility – no. (% denotes row percentage)**			
English	9513 (12.0%)	69,843 (88.0%)	0.298
French	205 (11.2%)	1627 (88.8%)	
**Language concordance/discordance between resident and LTC Facility – no. (% denotes row percentage)**			
Anglophone in English Facility	9244 (12.0%)	67,802 (88.0%)	0.14
Anglophone in French Facility	110 (12.8%)	747 (87.2%)	
Francophone in English Facility	269 (11.6%)	2041 (88.4%)	
Francophone in French Facility	95 (9.7%)	880 (90.3%)	

After adjusting for potentially confounding variables, the odds of hospitalization were 39% lower for residents with dementia when compared to residents without dementia (OR 0.61, 95% CI 0.57–0.65). The odds of hospitalization were lower for Francophones than Anglophones; however, this difference was not statistically significant (OR 0.91, 95% CI 0.81–1.03). Male sex, prescription of five or more medications, and number of comorbid chronic conditions were associated with higher odds of hospitalization, while prior residence in a rural area, very high health instability, and total dependence for activities of daily living were associated with lower odds of hospitalization in the multivariate regression model (See Supplementary Table 1, Additional file 2).

**Fig. 1:**
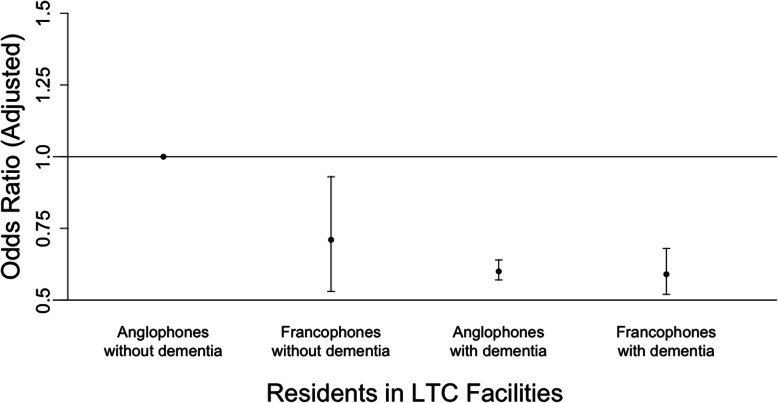
Effect modification of dementia and resident language on hospitalizations*. * Adjusted for age, sex, education, urban/rural status of prior residence, number of prescription medications, Charlson Comorbidity Index, CHESS score, Activities of Daily Living (ADL) scale) as well as facility characteristics (income quintile of facility, urban/rural status of facility, total number of beds

**Fig. 2:**
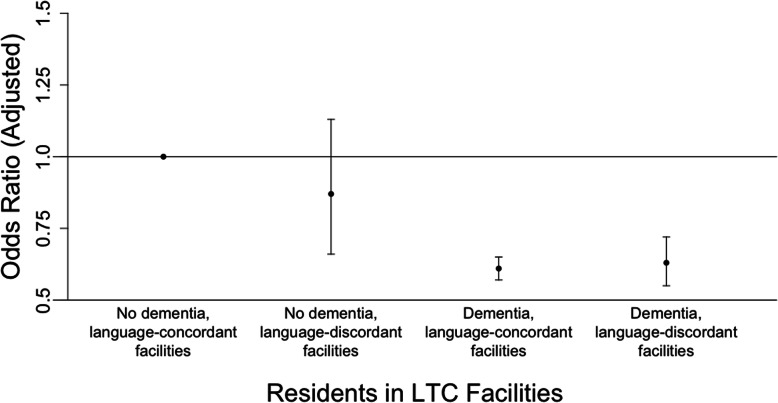
Effect modification of dementia and resident-facility language discordance on hospitalizations*. * Adjusted for age, sex, education, urban/rural status of prior residence, number of prescription medications, Charlson Comorbidity Index, CHESS score, Activities of Daily Living (ADL) scale) as well as facility characteristics (income quintile of facility, urban/rural status of facility, total number of beds

Next, we considered interactions between dementia and resident language (Fig. 1) as well as dementia and resident-facility language discordance (Fig. 2), while also controlling for confounding variables. The adjusted odds of hospitalization was greatest for Anglophones without dementia; as illustrated in Fig. 1, the odds ratio was significantly less than 1 for Anglophones with dementia (OR 0.60, 95% CI 0.57–0.64), Francophones without dementia (OR 0.71, 95% CI 0.53–0.93), and Francophones with dementia (OR 0.59, 95% CI 0.52–0.68) when compared to Anglophones without dementia. For residents without dementia, the adjusted odds of hospitalization did not differ significantly when comparing residents from language-discordant facilities to those from language-concordant facilities (OR 0.87, 95% CI 0.66–1.13). However, residents with dementia in both language-concordant and language-discordant facilities had lower odds of hospitalization when compared to residents without dementia in language-concordant facilities (Fig. 2).

## Discussion

In this study of LTC residents in Ontario, Canada, we found that residents with dementia were less likely to be hospitalized when compared to residents without dementia, even after adjusting for the significantly greater health stability and lower number of chronic conditions. Francophone residents had lower odds of hospitalization than Anglophone residents, but this difference was not statistically significant (OR 0.91, 95% CI 0.81–1.03). We investigated the interaction between dementia status and linguistic factors (resident language and resident-facility language discordance). For residents with dementia, the risk of hospitalization was not influenced by linguistic factors. However, the odds of hospitalization were 29% less for Francophones without dementia when compared to Anglophones without dementia. At the population-level, Francophones are more frequent users of long-term care; this finding has been attributed to the tendency of Francophones to live in less affluent neighbourhoods and in rural areas, where the community-based resources and services are limited [47]. It is possible that Francophones experienced a lower rate of hospitalization in this study because they lacked support (e.g., from family and friends, or financial) to assist them in transferring to and from hospitals.

Previous studies have shown that residents of LTC facilities with dementia have lower rates of hospitalizations than residents without dementia [28–30]. Several mechanisms have been proposed to explain this finding. First, individuals with dementia tend to have less aggressive end-of-life care goals; they are more likely to have “Do Not Resuscitate” and “Do Not Hospitalize” orders when compared to residents without dementia [28, 29]. Second, residents with dementia generally enter long-term care because of cognitive morbidity rather than physical morbidity. In our study, residents with dementia tended to have fewer chronic conditions and generally required less assistance with their activities of daily living. Since neurocognitive disorders are less amenable to medical and surgical treatments than physical conditions, it is possible that residents with dementia were less likely to be hospitalized as a result of the paucity of treatment options that could improve their quality of life or modify the course of their illness. Finally, hospitalizations can lead to a wide range of harmful events, such as delirium, falls, functional decline, iatrogenic complications, and infections [6–9]; since frail, elderly patients have an increased risk of experiencing in-hospital harm, the decision to admit a patient from a LTC facility must carefully balance the benefits and the risks, which can be significant.

The odds of hospitalization for residents without dementia was 13% less in language-discordant facilities than in language-concordant facilities; however, this difference was not statistically significant. We hypothesized that residents in language-discordant facilities would potentially experience a lower rate of hospitalization because of sub-optimal patient-provider communication, which is critical for the provision of quality care. If symptoms are not recognized or are underreported because of poor patient-provider communication, patients may potentially have fewer investigations and fewer hospitalizations. Since French facilities were defined as facilities required by law to provide services in both English and French, it is possible that Anglophones in French facilities did not experience many communication problems, thereby diminishing the magnitude of the effect of language discordance at the population-level.

We had also hypothesized that the communication deficits associated with cognitive impairment [11] would act synergistically with language discordance, but we found no such convincing effect. Some patients with dementia are not able to provide accurate information or reliable histories because of their condition; thus, healthcare providers may be more likely to obtain collateral information from caregivers or make inferences from objective tests [48, 49]. As a result, the impacts of poor patient-provider verbal communication may be attenuated for patients with dementia.

### Strengths and limitations

This study has many strengths, including its large population-based cohort and its use of validated datasets that allowed us to control for many potentially confounding variables. However, this study also had limitations. First, we did not have information pertaining to residents’ goals of care, which could have influenced the likelihood of hospitalization [50]. Also, we did not have information on whether the hospitalizations were clinically appropriate, which may have affected hospitalization rates, especially if some hospitalizations were avoidable or medically unnecessary [50]. Moreover, resident language was obtained from in-home assessments using RAI-MDS 2.0, a validated questionnaire for frail, elderly residents. Since interviewers may only record one language for each resident, it is unclear how language was assigned to patients who speak multiple languages. Furthermore, interviewers do not assess language proficiency. However, preliminary analyses performed by our group have shown that the language variable in the CCRS database has a high level of agreement (kappa = 0.76) with self-reported language spoken at home (Batista et al., unpublished data, 2019). Similarly, we were not able to control for language capacities of the LTC facilities (including interpreter use), nor were we able to determine the language used by health care providers when interacting with residents. We assumed that health care professionals in English and French facilities had a higher level of language competence in English and French, respectively. However, all LTC facilities may provide services in French, including those not required by law to do so. Thus, it is likely that some residents received care in their mother tongue even in settings that we deemed to be language-discordant. Finally, our findings may not be generalizable to residents who speak languages other than English or French, or those residing in regions outside Ontario or Canada, where legislation regarding language rights may be different.

## Conclusions

In conclusion, residents of LTC facilities with dementia were less likely to be hospitalized than residents without dementia. Overall, Anglophones and Francophones were equally likely to be hospitalized; however, Francophones were less likely to be hospitalized than Anglophones when considering the subgroup of residents without dementia. This finding could be due to poor patient-provider communication experienced by Francophone residents in LTC facilities, leading to fewer investigations and hospitalizations as a result of their symptoms being unrecognized by their healthcare providers. Further research is needed to investigate the complex interaction between linguistic factors and quality of care, especially among frail, elderly individuals with an increased risk of cognitive and communication deficits.

## Supplementary information

**Additional file 1.** Chronic conditions (Algorithm for chronic conditions).

**Additional file 2.** Predictors of hospitalization within 3 months of index assessment (The odds of hospitalization for residents with dementia compared to residents without dementia).

**Additional file 3.** Effect modification of dementia and resident language on hospitalizations (dementia*language) (Regression analysis denoting the interaction between dementia status and resident language).

**Additional file 4.** Effect modification of dementia and resident-facility language discordance on hospitalizations (dementia*discordance) (Regression analysis denoting the interaction between dementia status and resident-facility language discordance).

## Data Availability

The datasets used and/or analysed during the current study are available from the corresponding author on reasonable request. The dataset supporting the conclusions of this article is included within the article (and its additional files).

## Abbreviations

LTC: Long-term care
CCRS: Continuing Care Reporting System
RAI-MDS 2.0: Resident Assessment Instrument – Minimum Data Set 2.0
OHIP: Ontario Health Insurance Plan
ODB: Ontario Drug Benefit
RPDB: Registered Persons Database
DAD: Discharge Abstract Database
RAI-HC: Resident Assessment Instrument – Home Care
ANOVA: Analysis of variance
CHESS: Changes in Health, End stage disease, and Signs and Symptoms
CHF: Congestive Heart Failure
COPD: Chronic Obstructive Pulmonary Disease
OR: Odds Ratio
CI: Confidence Interval
