# Supplementary material for: The impact of dementia and language on hospitalizations: a retrospective cohort of long-term care residents
Source: BMC Geriatr. 2020 Oct 8;20:397. doi: 10.1186/s12877-020-01806-2 (PMC7545542; doi:10.1186/s12877-020-01806-2)
Supplement: Supplementary file 1 — Additional file 1. Chronic conditions (Algorithm for chronic conditions). [file 12877_2020_1806_MOESM1_ESM.docx]

**Additional file 1. Chronic conditions**

We identified a total of 18 chronic conditions using administrative data from April 1, 2001 onwards.

We used validated algorithms to ascertain cases of the following 8 chronic conditions:

1. Acute myocardial infarction
2. Asthma
3. Congestive Heart Failure
4. COPD
5. Dementia
6. Diabetes
7. Hypertension
8. Rheumatoid Arthritis

The remaining 10 chronic conditions were defined according to inpatient hospital diagnostic codes (at least 1 from Discharge Abstract Database) or outpatient physician billing codes (at least 2 from Ontario Health Insurance Plan within a 2-year period):

1. Arrhythmia
2. Cancer
3. Coronary Heart Disease
4. IBD
5. Non-psychotic mood and anxiety disorders
6. Osteoarthritis
7. Osteoporosis
8. Other mental health conditions
9. Renal Disease
10. Stroke
