# Supplementary material for: The impact of dementia and language on hospitalizations: a retrospective cohort of long-term care residents
Source: BMC Geriatr. 2020 Oct 8;20:397. doi: 10.1186/s12877-020-01806-2 (PMC7545542; doi:10.1186/s12877-020-01806-2)
Supplement: Supplementary file 4 — Additional file 4. Effect modification of dementia and resident-facility language discordance on hospitalizations (dementia*discordance) (Regression analysis denoting the interaction between dementia status and resident-facility language discordance). [file 12877_2020_1806_MOESM4_ESM.docx]

**Additional file 4**: **Supplementary table 3.** Effect modification of dementia and resident-facility language discordance on hospitalizations (dementia*discordance)

| **Coefficient** | **Multivariate Adjusted Model, OR (95% CI)** |
| --- | --- |
| **Language discordance and dementia status**  Discordance, Dementia  Discordance, No Dementia  No Discordance, Dementia  No Discordance, No Dementia (ref) | 0.63 (0.55 – 0.72)  0.87 (0.66 – 1.13)  0.61 (0.57 – 0.65)  1.00 (ref) |
| **Age (coefficients for restricted cubic spline)***  Age  (Age – 82)^3  (Age – 87)^3  (Age – 91)^3 | 0.99 (0.98 – 1.00)  0.97 (0.93 – 1.01)  1.33 (0.95 – 1.85)  0.47 (0.21 – 1.03) |
| **Sex**  Female (ref)  Male | 1.00 (ref)  1.40 (1.34 – 1.47) |
| **Rurality**  Urban (ref)  Rural  Missing | 1.00 (ref)  0.92 (0.86 – 0.98)  0.77 (0.67 – 0.89) |
| **CHESS**  No Health Instability (ref)  Minimal Health Instability  Low Health Instability  Moderate Health Instability  High Health Instability  Very High Health Instability | 1.00 (ref)  1.31 (1.25 – 1.38)  1.41 (1.32 – 1.51)  1.65 (1.51 – 1.81)  1.48 (1.24 – 1.75)  0.59 (0.38 – 0.93) |
| **ADL hierarchy**  Independent (ref)  Supervision Required  Limited Impairment  Extensive Assistance Required (1)  Extensive Assistance Required (2)  Dependent  Total Dependence | 1.00 (ref)  1.01 (0.85 – 1.20)  1.19 (1.03 – 1.38)  1.09 (0.95 – 1.26)  1.14 (0.98 – 1.31)  1.03 (0.89 – 1.20)  0.78 (0.66 – 0.93) |
| **Drug subclass**  <5 Subclasses (ref)  5-9 Subclasses  10+ Subclasses | 1.00 (ref)  1.18 (1.07 – 1.31)  1.47 (1.33 – 1.64) |
| **Income quintile**  5 (ref)  4  3  2  1  Missing | 1.00 (ref)  0.98 (0.88 – 1.10)  0.93 (0.82 – 1.06)  1.11 (0.98 – 1.26)  1.02 (0.91 – 1.15)  1.87 (1.44 – 2.42) |
| **Highest level of education**  University Graduate (ref)  Some post-secondary  High School  Less Than High School  Unknown | 1.00 (ref)  0.97 (0.87 – 1.07)  0.96 (0.87 – 1.06)  0.99 (0.91 – 1.09)  1.05 (0.96 – 1.14) |
| **Total number of beds** | 1.00 (1.00 – 1.00) |
| **Number of prevalent conditions**  0 (ref)  1  2  3  4  5  6  7  8 | 1.00 (ref)  1.14 (0.92 – 1.42)  1.22 (0.99 – 1.50)  1.37 (1.12 – 1.67)  1.61 (1.31 – 1.97)  1.77 (1.44 – 2.17)  1.98 (1.62 – 2.43)  2.12 (1.72 – 2.62)  2.51 (2.02 – 3.11) |
| **Chronic conditions**  No chronic condition  Cancer  CHF  COPD  Diabetes | 1.00 (ref)  1.10 (1.04 – 1.17)  1.28 (1.21 – 1.35)  1.06 (1.01 – 1.12)  1.01 (0.97 – 1.06) |
| * Restricted cubic spline with 5 knots chosen at the following percentiles: 5 (71), 27.5 (82), 50 (87), 72.5 (91), 95 (97).  Note: CHF = Congestive Heart Failure; COPD = Chronic Obstructive Pulmonary Disease; OR = Odds Ratio | |
